# Salt‐Induced Modulation of Self‐Assembly in C8‐10 AlkylPolyGlucoside/Fatty Alcohol Formulations

**DOI:** 10.1002/cplu.202500390

**Published:** 2025-08-19

**Authors:** Lorenzo Veronico, Luigi Gentile

**Affiliations:** ^1^ Department of Chemistry University of Bari Aldo Moro via Orabona 4 70125 Bari Italy; ^2^ Center for Colloid and Surface Science (CSGI) via della Lastruccia 3 50019 Florence Italy

**Keywords:** APGs, bio‐based surfactants, brine, formulation, SAXS

## Abstract

A formulation strategy based on the alkyl polyglucoside (APG) surfactant Triton CG‐110, in combination with 1‐Dodecanol as a co‐surfactant and brine as an additive, is developed. The results highlight the pivotal role of brine in modulating the rheological properties of the system, inducing a shift from viscous to viscoelastic and yield‐pseudoplastic behavior at elevated surfactant and salt concentrations. Small‐angle X‐ray scattering analysis reveals that increased salinity enhances interparticle correlations within bicellar nanostructures, which are closely associated with improved viscoelasticity and formulation stability. The salting‐out effect, combined with the ability to incorporate 1‐Dodecanol, leads to nanoscale structural transitions that provide a rational basis for tuning the performance of surfactant systems. The resulting high oil‐uptake efficiency of 82% in the final formulation demonstrates a viable pathway toward high‐efficiency, environmentally benign surfactants, supporting the broader transition to greener chemistries in industrial applications.

## Graphical abstract

Salt's effect on the APG:1‐Dodecanol system was studied. Small‐angle X‐ray scattering showed salt increased interparticle correlation, leading to higher structural organization at higher salt concentrations. This correlates with observed viscoelastic properties. Cleaning efficiency tests were also conducted on samples.

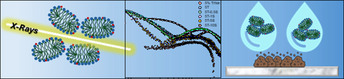

## 1 Introduction

Oil uptake and remediation have been longstanding global concerns due to the frequent occurrence of devastating environmental events. It is imperative to develop effective solutions for mitigating and addressing these issues.^[^ ^1^ ^,^, ^2^ ^]^ One of the easiest approach involves the use of surfactants, which are amphiphilic molecules capable of solubilizing organic compounds by incorporating them into micelles.^[^ ^3^, ^4^, ^5^ ^]^ However, the production and utilization of surfactants present significant environmental concerns,^[^ ^6^ ^]^ primarily due to their reliance on fossil‐derived sources and the industrial processes involved, which are often harmful to the environment.^[^ ^7^ ^]^ As a result, there is a pressing need for the development of more sustainable alternatives. Recent scientific advancements have introduced promising surfactants, such as alkyl polyglycosides (APGs), rhamnolipids, and sophorolipids^[^ ^8^ ^,^, ^9^ ^]^ that are designed to be biodegradable, breaking down into nontoxic byproducts.^[^ ^10^ ^]^ Rhamnolipids and sophorolipids, classified as biosurfactants, represent the most environmentally friendly alternatives, as they are entirely produced by living organisms—bacteria and yeasts, respectively.^[^ ^11^, ^12^, ^13^ ^]^ In terms of performance, these biosurfactants are comparable to traditional surfactants.^[^ ^14^ ^]^ However, their high production costs currently limit their widespread use.^[^ ^15^ ^]^ This limitation is further compounded by their inherent structural complexity, which, despite their promising performance, fundamentally restricts their broader application. In contrast, APGs are bio‐based nonionic surfactants^[^ ^16^ ^]^ with low molecular weight,^[^ ^17^ ^]^ usually synthesized through a glycosidation reaction involving a sugar and a fatty alcohol.^[^ ^18^ ^]^ While their synthesis involves a certain complexity, their overall production cost is notably lower compared to biosurfactants like rhamnolipids and sophorolipids. Hence, they are not only more easily available than biosurfactants but they can be well modulated in diverse application,^[^ ^19^ ^,^, ^20^ ^]^ often demonstrating performance on par with petrochemical analogs. In water solutions, APGs first aggregate into micelles at the critical micelle concentration (CMC), and this micellar phase region is usually large. APGs may self‐assembly into micelles of various shapes.^[^ ^21^ ^]^ The compounds with C_8_–C_10_ alkyl chains form spherical (ball)‐ or disc‐shaped micelles at concentrations above 7 × 10^−3^% (w/w). When the concentration increases, the number of aggregates also increases, micelles become deformed, and, at a concentration of about 5 × 10^−2^% (w/w), they become cylinder‐ like. An increase in the chain length to C_12–14_ results in a significant drop in CMC and an increase in the length of the cylinder‐shaped micelles.^[^ ^16^ ^]^The phase behavior of simple APG/water binary systems is only slightly influenced by temperature. APGs do not exhibit a cloud point at elevated temperatures in the presence of salts. This property renders them particularly suitable for high‐temperature and high‐salinity reservoirs, where conventional surfactants tend to adsorb onto carbonate surfaces.^[^ ^22^ ^]^ The APG in Triton CG‐110 is D‐glucopyranose oligomeric decyl octyl glycoside (C_8–10_G_1–2_) and presents a CMC of 1748ppm (≈0.17 v/v%) at a temperature of 25 °C.^[^ ^5^ ^]^ While Triton CG‐110 typically forms spherical micelles, its co‐formulation with 1‐Dodecanol induces a morphological transition to a bicellar phase, finally, further increases in surfactant concentration facilitate a subsequent phase transition from the bicellar to a lamellar phase. The emulsifying power generally depends on the length and nature of the hydrophobic part of the surfactant. It was shown that the stability of the emulsion formed increases with the increase of alkyl chain length. With an increasing alkyl chain length, the solubility of the surfactant in the oil phase increases, forming a highly stable emulsion. Additional testing has proved that APG formulations can provide interfacial properties that are mostly independent of both salinity and temperature.^[^ ^20^ ^]^ To enhance surfactant efficiency usually co‐surfactants and additives are added to the formulation. Fatty alcohols have historically been incorporated into surfactant formulations as co‐surfactants across a wide range of applications.^[^ ^23^, ^24^, ^25^, ^26^ ^]^ A thorough investigation into the influence of long‐chain fatty alcohols in combination with the cationic surfactant cetyltrimethylammonium chloride (CTAC) has demonstrated that the final structural and physicochemical properties of the resulting mixtures are highly dependent on the cooling rate applied following high‐temperature preparation.^[^ ^25^ ^,^, ^27^ ^]^ Notably, *Miyake and Einaga* examined the role of 1‐Dodecanol in the polyoxyethylene alkyl ether–water system, demonstrating its ability to induce the formation of worm‐like micelles.^[^ ^26^ ^]^ Incorporation of non‐polar co‐solvents, such as fatty alcohols, is a widely employed strategy to tailor interfacial tension, viscoelasticity,^[^ ^28^ ^]^ and improve oil uptake efficiency.^[^ ^29^, ^30^, ^31^ ^]^ Such additives can induce significant micellar restructuring, often driving transitions from spherical to elongated rod‐like aggregates with increasing concentration. C_16–18_ APG with Cetearyl glucoside and Cetearyl alcohol was investigated as emulsifier formulation where the formation of a mosaic texture lamellar phase was demonstrated.^[^ ^32^ ^]^ More recently, Triton CG‐110 was investigated in presence of 1‐Dodecanol indicating the presence of bicelles at low surfactant concentration in the specific ratio of 2.56:1.^[^ ^33^ ^]^ Furthermore, on the application point of view, fatty alcohols have been recognized for their capacity to enhance the oil recovery efficiency of Alkyl PolyGlucoside (APG) surfactants.^[^ ^5^ ^]^

For ionic APGs, when salt is added to the system, ionic strength primarily governs electrostatic interactions. Increasing salinity screens the charge on the surfactant headgroups, reducing electrostatic repulsion between micelles and potentially leading to aggregation or changes in micelle shape.^[^ ^34^ ^,^, ^35^ ^]^ Conversely, for nonionic APGs such as Triton CG‐110, the "salting‐out" effect becomes more prominent.^[^ ^36^ ^]^ These observations reveal intrinsic formulation challenges for APGs, notably under varying salinity levels where their stability and functional properties may be considerably compromised. Moreover, the salt effect in presence of a fatty alcohol co‐surfactant was not investigated in our knowledge.

This study systematically investigates the brine effect on a Triton CG‐110 (Triton)‐1‐Dodecanol (C_12_OH) formulation. The mechanical properties of the formulated systems were characterized *via* rheological analysis, and their structural organization was probed using small‐angle X‐ray scattering (SAXS). The findings elucidate the impact of brine addition on the physicochemical properties of surfactant‐fatty alcohol systems, thereby providing novel insights crucial for optimizing detergent and emulsion formulations. Moreover, the oil absorption efficiency was determined.

## 2 Results and Discussion

### 2.1 Formulation of the Samples

Samples were prepared with varying concentrations of Triton, labeled with T and salt, labeled with S, maintaining a constant Triton‐to‐1‐Dodecanol ratio. Full details on sample preparation and percentage compositions are provided in the Experimental Section.

### 2.2 Effect of Brine on C_8–10_G_1–2_ – C_12_OH Formulation at 25 °C

**Figure 1:**
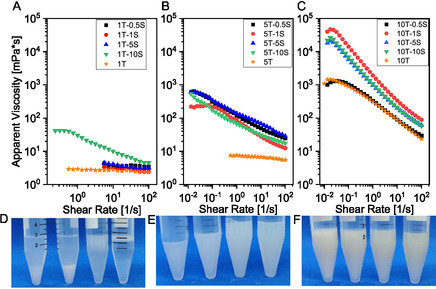
Flow curve test of the samples in Table 1 in panel A,B, and C, respectively for 1T, 5T, and 10T series. Samples stability after 48 h in panel D,E,F, respectively for 1T, 5T,and 10T series.

The effect of salinity into the Triton‐C_12_OH system induces significant effects, as evidenced by flow curve test as a function of formulation and brine concentration (**Figure** **1**). The system exhibits three distinct rheological regimes contingent upon the salt concentration. At low salt levels (Figure 1A), the apparent viscosity profile of the formulations remain largely unaffected up to approximately 10 v/v%, beyond which a slight shear‐thinning behavior becomes apparent.

At intermediate Triton concentrations (Figure 1B), a moderate rheological response is observed across all formulations, characterized by a mild shear‐thinning profile.

In contrast, at higher surfactant concentrations (Figure 1C), the influence of salt is markedly enhanced, resulting in a yield pseudoplastic behavior (**Table** **1**). This phenomenon could be attributed to the salting‐out effect, where salt ions preferentially hydrate, effectively competing with surfactant molecules for water. These systems often exhibit shear‐thinning behavior, a critical characteristic for applications that require mobility control.^[^ ^36^ ^]^ Among the tested formulations, sample 10T‐1S emerges as having the optimal rheological characteristics. This could be attributed to its higher surfactant concentration, which enhances structural packing and network formation. This observation highlights a nonlinear relationship between salt concentration and system properties: an initial increase in salt improves rheological performance up to a maximum—likely by promoting tighter packing—beyond which further salinity induces the salting‐out effect, resulting in a deterioration of properties and eventual precipitation.

**Table 1:** Yield stress and flow index value determined with the Herschel‐bulkley model of the data in Figure 1C.

Sample	Yield Stress/Pa	Flow index
10T‐1S	1.0	0.489
10T‐5S	0.49	0.503
10T‐10S	0.42	0.536

The colloidal stability of each series was evaluated over a 48‐hour period by visual inspection. In the case of the 1T series, complete phase separation was observed across all examined salt concentrations, indicating poor stability (Figure 1D). Conversely, both the 5T and 10T series exhibited sustained colloidal stability throughout the duration of the study and remained stable beyond the 48‐hour timeframe (Figure 1E,F). The salting‐out reduces surfactant hydration, which can induce phase separation or precipitation as reported in literature.^[^ ^37^ ^]^ The phase behavior presented in Figure 1D suggests two distinct manifestations of phase separation; however, both phenomena are fundamentally driven by the salting‐out effect. Specifically, samples 1T‐0.5S and 1T‐1S (left side of Figure 1D) exhibit rapid phase separation. This is likely due to their relatively low salt concentrations, which are comparable to or slightly below the surfactant concentration, leading to an immediate and pronounced salting‐out effect. Conversely, samples 1T‐5S and 1T‐10S, characterized by significantly higher salt concentrations relative to the surfactant, probably form kinetically more stable temporary structures that delay the full phase separation. A discernible trend is evident in the Figure 1D, with sample 1T‐5S demonstrating an intermediate precipitation behavior between that observed for 1T‐1S and 1T‐10S.

**Figure 2:**
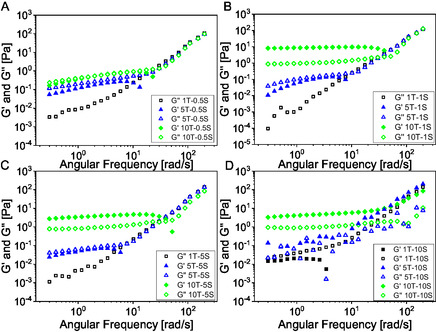
Frequency sweep test of the samples in Table 1. In figure are reported G′ and G′′; as function of surfactant concentration, while maintaining salt concentration fixed A) at 0.5 wt%, B) at 1 wt%, C) at 5 wt%, and at D) 10 wt%.

Frequency sweep measurements allow a more comprehensive understanding of the influence of brine on the rheological behavior of the Triton‐C_12_OH system. **Figure** **2** illustrates the impact of four different salt concentrations across varying surfactant concentrations.

In the 1T series, only the loss modulus (G″) is observed across all salt concentrations, indicative of a predominantly viscous, liquid‐like behavior. This observation confirms the findings from the flow curve tests where a Newtonian‐like behavior was observed. For the 5T series, the weak shear‐thinning behavior noted in the flow curves is further supported by the trends in both the storage (G′) and loss moduli obtained from the frequency sweep analysis. At low angular frequencies, G′ and G″ are nearly superimposed, indicating a weakly pseudoplastic and non‐Newtonian character.

In contrast, the 10T series exhibits a predominance of the storage modulus over the loss modulus across most angular frequency values and salt concentrations, indicating a shift toward more elastic, structured behavior. An exception is observed for the 10T‐0.5S sample, where G′ and G″ are nearly equivalent at low frequencies (Figure 2A). These findings align with the yield‐pseudoplastic profiles observed in the corresponding flow curve tests,

At higher salt concentrations, despite relatively low absolute modulus values, the samples exhibit the typical characteristics of gel‐like behavior. There are two main mechanism that can explain this behavior. The salting‐out effect were salts compete with surfactant headgroups for water, effectively reducing hydration, promoting tighter packing and favorable micelle growth. In the presence of salts, C_12_OH incorporation into micelles becomes more favorable, which help stabilizing larger, more flexible self‐assembled structures. The viscoelasticity could be due to the formation of the so‐called worm‐like micelles.^[^ ^38^ ^]^ In fact, this long, flexible micelles form elongated networks responsible for viscoelastic behavior. However, worm‐like micellar system typically exhibit a quasi‐Maxwellian response in frequency sweep measurements.^[^ ^39^ ^,^, ^40^ ^]^ Such behavior was not observed in our case, suggesting that the system does not fully conform to the rheological signature of worm‐like micelles.

### 2.3 Oil‐Uptake Capability

**Figure 3:**
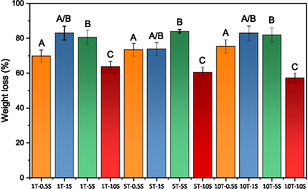
Oil‐uptake efficiency of the 0.5S series (orange), 1S series (blue), 5S series (green), and 10S series (red). Superscript letters above each column denote statistically homogeneous groups in terms of weight percent removed. These groupings were determined via one‐way analysis of variance (ANOVA), followed by Tukey's post‐hoc test at a 5% significance level, using salt concentration as the grouping variable.

To evaluate the oil‐uptake capability of the formulations investigated the Industrieverband Körperpflege‐ und Waschmittel (IKW) test was conducted. The IKW test is an industrial standard used to assess cleaning efficiency. In the IKW test, the practical measure of “cleaning” largely corresponds to oil uptake or removal of oily contaminants. For this reason, in this study, oil uptake and cleaning performance are used interchangeably to describe the surfactant system's effectiveness in removing oil contaminants. The cleaning performance, evaluated in terms of either weight loss or oil uptake, is presented in **Figure** **3**. To assess the statistical significance of differences among the four sample sets, each prepared at a distinct salt concentration, a one‐way Analysis of Variance (ANOVA) was performed. Following ANOVA, Tukey's post‐hoc test was applied at a 5% significance level to identify specific group differences. This analysis, which assigned alphabetical labels to the sample sets for clarity, revealed the presence of three statistically distinct and internally homogeneous groups, in addition to a fourth group demonstrating intermediate characteristics between those identified as Group A and Group B. Among all tested formulations, 5T‐5S exhibited the highest overall cleaning efficiency. Notably, in the absence of added salt, the cleaning performance decreased with increasing surfactant concentration, yielding efficiencies of 57% for 1T, 37% for 5T, and 34% for 10T, respectively.^[^ ^5^ ^]^ For reference, a commercial soap containing 10% SDS yielded significantly lower efficiencies across the board: 20% at 1, 16% at 5, and 19% at 10 v/v%.^[^ ^4^ ^]^ Notably, all formulations incorporating salt demonstrated cleaning performances either equivalent to or surpassing their salt‐free counterparts. These results consistently outperform both the commercial benchmark and recently developed alternative systems.

### 2.4 Structural Investigation

SAXS data for a 5 v/v% Triton in water can be well modeled using a core–shell sphere form factor, consistent with the formation of spherical micelles. Upon the introduction of C_12_OH, the system exhibits a transition to a bicelle‐like structure.^[^ ^5^ ^]^

Considering an average headgroup area of 72.5 Å^2^, an average tail volume of 269.5 Å^3^, and an average tail length of 12.9 Å leads to an estimated critical packing parameter (CPP) of 0.29, which confirms the experimental observation of spherical micelles. Introducing C_12_OH at a ratio of C_8–10_G_1–2_ to fatty alcohol of 2.56:1 results in an averaged CPP of 0.33. The value of 0.33 just reaches the lower boundary of the typical worm‐like micelle formation range. Literature indicates that worm‐like micelle formation usually requires CPP values around 0.4–0.5 or higher, often achieved by increasing tail volume, reducing headgroup area, or temperature and salt conditions that affect effective molecular geometry.^[^ ^33^ ^,^ ^41^ ^]^

**Figure 4:**
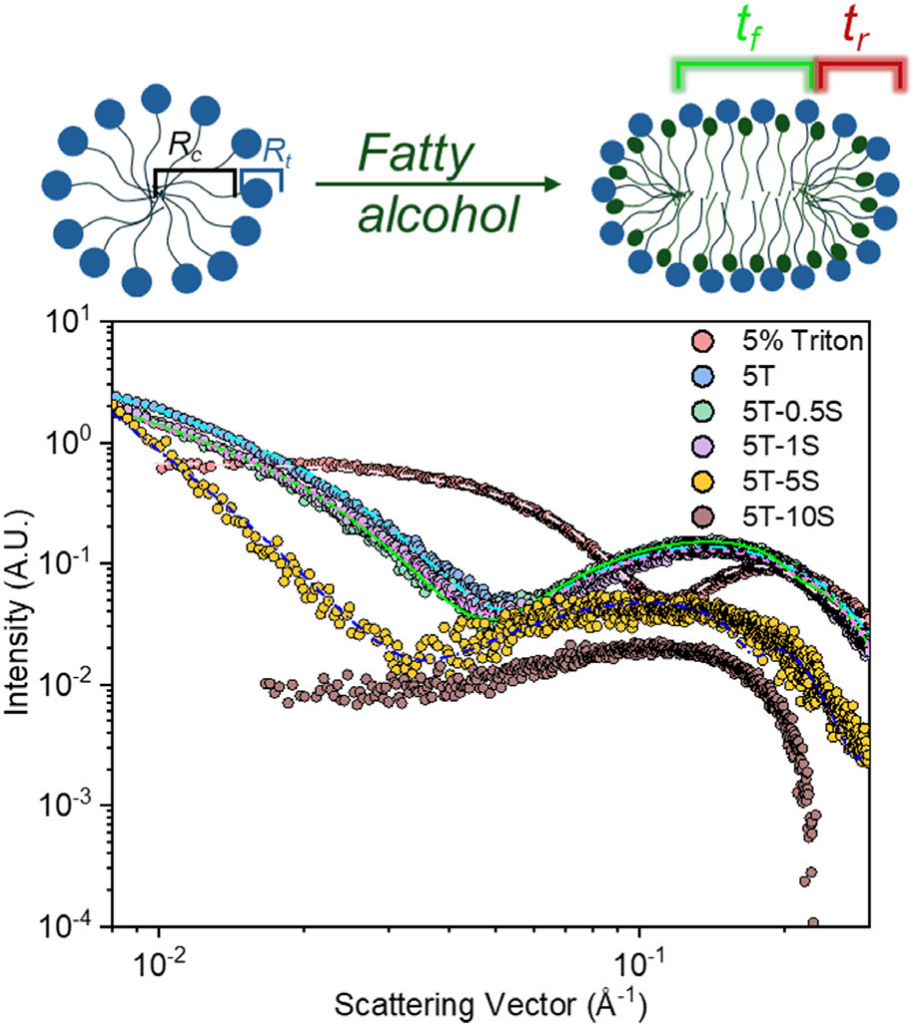
Above, a scheme representing the micelle‐to‐bicelle transition with the addition of 1‐dodecanol. Below, best‐fit curves represent different models: a core–shell sphere model for 5% Triton (light pink dashed line). All other samples are modeled with a core–shell bicelle form factor incorporating a sticky hard sphere structure factor: 5T (light blue dash‐dot), 5T‐0.5S (green line), 5T‐1S (purple short dash), and 5T‐5S (blue short dash‐dot). For sample 5T‐10S, only the data are shown.

The addition of NaCl up to ≈1 wt% does not produce significant changes in the SAXS profile (**Figure** **4**). At NaCl concentrations of 5 and 10 wt%, a broader scattering feature appears in the mid‐*q* region, corresponding to the form factor of thin disk‐like structures. This feature arises from the contrast in electron density between the hydrocarbon‐rich bicelle core and the surrounding solvent and is typically associated with the bicelle thickness. At the same time, the scattering intensity in the low‐*q* regime (0.008–0.03 Å^−^^1^) diminishes significantly. These observations lead us to conclude that the origin of the bump may partly arise from structure factor effects overlapping with the form factor, as seen in similar systems.^[^ ^34^ ^]^ The emergence of a broadened SAXS bump at mid‐*q*, influenced by sticky hard sphere structure factor contributions, reflects enhanced interparticle correlations among bicelles under high salt conditions. In the case of the 5T‐10S, the structure factor broadened the bump in a way that makes it challenging to accurately model the system.

This increased spatial organization is consistent with the development of weakly elastic features in the system, as evidenced by the appearance of a finite storage modulus (G′) in the frequency sweep. The system thus transitions from a viscous bicellar solution to a viscoelastic bicellar dispersion, wherein transient inter‐bicelle interactions contribute to elastic energy storage. The parameters obtained by the fitting Equations (see Equation S1, S2, and S3, Supporting Information) are reported in **Table** **2**.

**Table 2:** SAXS fitting parameters obtained for 5 v/v% triton solution, 5T, 5T‐0.5S, 5T‐1S, 5T‐5S, and 5T‐10S. For 5 v/v% triton solution, a core–shell sphere model PCSS(q) was applied. For all the other investigated samples a core–shell bicelle PCSB(q) model with a sticky hard sphere S(q) was applied. The estimated uncertainty is around ±0.3 Å.

	5% Triton	5T	5T‐0.5S	5T‐1S	5T‐5S
Radius, *R* or $R_{c}$ [Å]	14.8	18.8	19.3	18.7	23.9
Core‐Shell, $R_{t}$ [Å]	9.1	–	–	–	–
Rim tickness, *t*_R_ [Å]	–	11.0	10.7	10.4	12.1
Face tickness, *t*_F_ [Å]	–	5.0	4.0	4.0	6.0
Length, *L* [Å]	–	527	512	560	606
Effective Radius, *R*_eff_ [Å]	–	17.79	18.19	17.5	21.8
Stickiness	–	0.021	0.021	0.022	0.023

## 3 Conclusion

Brine addition affects the Triton‐C_12_OH system at the nanoscale leading to a microscopic mechanical effect. In fact, in the rheological behavior, a transition from primarily viscous, at low surfactant and salt levels, to significantly viscoelastic as both concentrations increase was noticed. Higher surfactant content combined with salt leads to a pronounced yield‐pseudoplasticity, indicating enhanced internal structure. High salt concentration reduce the hydration of sugar headgroups lowering the solubility of the surfactant in water (salting‐out) promoting precipitation. In this conditions C_12_OH would not be stabilized by the surfactant contribution to the precipitation process. SAXS analysis reveals the structural insights of the system. A transition from spherical micelles to bicelle‐like structures upon C_12_OH addition. Moreover, higher salt concentrations induce changes in the scattering profile, indicative of enhanced interparticle correlations and the formation of organized, thin disk‐like bicelles. This increased structural organization at higher salt concentrations directly correlates with the observed development of viscoelastic properties and improved colloidal stability at mid concentration systems, suggesting that salt‐induced interparticle attractions drive the formation of a transient network. From the application point of view, this enhanced stability coupled with the observed improvements in oil‐uptake capabilities demonstrated by the IKW test, where salt‐containing formulations consistently outperformed their salt‐free counterparts and commercial standards, highlights the practical benefits of brine inclusion. The increased oil uptake can be attributed to the formation of larger and more structurally organized aggregates, which possess a greater capacity for oil incorporation and retention. These findings provide valuable insights into the design principles for developing high‐performance, surfactant‐based formulations for various applications.

## 4 Experimental Section

4.1

4.1.1

##### Chemicals

Triton CG‐110, a commercially available surfactant formulation, was acquired from Sigma–Aldrich (Darmstadt, Germany). This formulation comprises a blend of 58.0–62.0 wt% D‐glucopyranose oligomeric decyl octyl glycoside (C_8–10_G_1–2_) and 38.0–42.0 wt% water. The degree of oligomerization, specifically the number of pyranose units present, was determined using proton nuclear magnetic resonance (^1^H‐NMR) spectroscopy (Figure S1, Supporting Information). Additionally, thermogravimetric analysis (TGA) was employed to assess the water content, which was found to be approximately 36 wt%. Based on these findings, Triton CG‐110 can be considered to consist predominantly of C_8–10_G_1–2_, and for clarity, the surfactant will be referred to as such in this study.

The critical micelle concentration (CMC) of Triton CG‐110 has been reported as 1748 ppm (≈0.17% v/v)^[^ ^5^ ^]^ at 25  °C, indicating its surfactant efficiency under ambient conditions. In addition to Triton CG‐110, 1‐Dodecanol and sodium chloride was procured from Sigma–Aldrich for experimental use. All raw materials were utilized as received, without any further purification, to maintain consistency with their commercially available formulations.

##### Samples Preparation

The preparation of samples involved the systematic blending of Triton with distilled water, sodium chloride, and 1‐Dodecanol. Specifically, two distinct sets of samples were formulated. In the first Triton was mixed with water at 1, 5, and 10% v/v resulting in C_8–10_G_1–2_ surfactant concentration of 0.64, 3.2, and 6.4% v/v relative to the initial solution. Furthermore, these Triton‐water mixtures were supplemented with 1‐Dodecanol at concentrations of 0.25, 1.25, and 2.5% v/v, establishing a C_8–10_G_1–2_ to fatty alcohol ratio of 2.56:1. The resulting ternary systems were subsequently combined with sodium chloride at varying concentrations of 0.5, 1, 5, and 10% wt%.

All the investigated samples were mixed by using a rheometer ensuring precise temperature control and shear mixing.^[^ ^5^ ^,^, ^27^ ^]^ Samples were mixed at 70 °C applying a shear rate of 1000s^−1^. Subsequently, the system was cooled down at a rate of 1°C/min while maintaining a shear rate of 100 s^−1^.

##### Stationary and Oscillatory Rheology

The rheological properties of the samples detailed in **Table** **3** were systematically investigated using a MCR302 Evolution stress‐controlled rheometer (Anton Paar GmbH, Graz, Austria). A concentric cylinder (Taylor–Couette) geometry was employed for all measurements, consisting of an inner cylinder with a diameter of 16.662 mm and a defined gap of 0.704 mm. Precise temperature control during the experiments was achieved by a Peltier system, maintaining the temperature at 25 °C with an accuracy of ±1 °C. An external water‐bath circulator was utilized to ensure stable thermal conditions.

**Table 3:** First set samples under investigation.

Label	Triton CG‐110[% v/v of the initial solution]	C_8–10_G_1–2_[% v/v]	C_12_OH[% v/v]	NaCl [% m/m]
1T	1	0.64	0.25	0
1T‐0.5S	1	0.64	0.25	0.5
1T‐1S	1	0.64	0.25	1
1T‐5S	1	0.64	0.25	5
1T‐10S	1	0.64	0.25	10
5T	5	3.2	1.25	0
5T‐0.5S	5	3.2	1.25	0.5
5T‐1S	5	3.2	1.25	1
5T‐5S	5	3.2	1.25	5
5T‐10S	5	3.2	1.25	10
10T	10	6.4	2.5	0
10T‐0.5S	10	6.4	2.5	0.5
10T‐1S	10	6.4	2.5	1
10T‐5S	10	6.4	2.5	5
10T‐10S	10	6.4	2.5	10

Rheological characterization was performed using both steady‐state and oscillatory shear experiments to investigate the flow behavior and viscoelastic properties of the 1T, 5T, and 10T samples as a function of salt concentration.

Steady‐state flow experiments were conducted by systematically increasing the shear rate $\dot{\gamma }$ and measuring the corresponding apparent viscosity $\eta (\dot{\gamma })$.

To further elucidate the viscoelastic characteristics of the systems, oscillatory shear experiments were performed. Initially, amplitude sweep tests were carried out by applying an oscillatory deformation at a fixed angular frequency while varying the strain amplitude logarithmically. This method allows for the determination of the linear viscoelastic regime (LVE), where the material response is proportional to the applied stress or strain. Within the LVE, the complex shear modulus G∗(*ω*) is measured, which comprises the storage modulus G′(*ω*), representing the elastic component, and the loss modulus G′′(*ω*), representing the viscous component as shown in the following equation:

(1) $$G^{*}\left(\omega \right)=G\prime \left(\omega \right)+iG\prime \prime \left(\omega \right)$$

After the amplitude sweeps, frequency sweep tests were performed within the previously determined LVE. These tests involved applying a fixed strain amplitude while varying the angular frequency (*ω*) logarithmically over a range typically from 10^−1^ to 10^2^ rad/s. Frequency sweeps provide detailed information on the frequency dependence of (G′) and (G′′), offering insights into the characteristic relaxation dynamics of the materials.

##### Cleaning Efficiency Test for Oil Uptake

To assess cleaning efficiency, an industrial standard known as the IKW (Industrieverband Körperpflege‐ und Waschmittel) was utilized as a reference [42]. This standardized test method is designed to evaluate the effectiveness of hard surface cleaners in removing stubborn, polymerized grease baked onto stainless steel plates. The experimental procedure involved the preparation of a sample of greasy soil, which was applied to a stainless steel tile and subsequently subjected to thermal aging at 135 °C for 2 h prior to its use in the cleaning efficacy evaluation. The stainless steel tiles were pre‐treated with a solution consisting of 0.08 wt./vol % potassium hydroxide and ethanol before contamination. The greasy soil was a mixture of oils and soil in a 4:1 ratio. Specifically, the oil component comprised peanut oil, sunflower oil, and corn oil, while the HSW soil component primarily consisted of humus, cement, silica gel, and clay. The contaminated stainless steel tiles were then placed in Petri dishes and immersed in pollutant‐removal solutions for 18 h. The tiles were weighed using an analytical balance both before and after exposure to the cleaning solutions to assess their effectiveness. Oil uptake was quantified using the following equation:

(2) $$\text{Weight}\mathrm{loss}\%=100\times \frac{W_{\mathrm{in}}-W_{f}}{W_{\mathrm{in}}-W_{p}}$$

where *W*_f_ denotes the final weight of the contaminated stainless‐steel tiles after 18 h of treatment with the test formulation, *W*_in_ corresponds to the initial weight of the contaminated tiles, and *W*_p_ represents the weight of the clean, uncontaminated tiles.

##### Statistical Analysis

Statistical analysis was performed using one‐way analysis of variance (ANOVA), followed by Tukey's post hoc multiple comparison test to assess the significance of differences between groups. All data were processed using MATLAB software (MathWorks, Natick, MA, USA). A confidence level of 95% (*p* < 0.05) was adopted to determine statistical significance.

##### Small‐Angle X‐Ray Scattering (SAXS)

SAXS measurements were carried out using an Anton Paar SAXSpoint 2.0 instrument (Anton Paar GmbH, Graz, Austria), equipped with a Primux 100 Micro source Cu K‐alpha X‐ray beam with a wavelength of 0.15418 nm and power of 50 W. The scattering intensity *I(q)* was recorded with a 75 × 75 ym^2^ pixel size detector Dectris Eiger R 1 M (Dectris Ltd, Baden, Switzerland). The sample holder was positioned at two sample‐to‐detector distance of 800 and 1050mm from the detector providing access to an overall scattering vector range of 0.008 Å^−^^1^ < *q* < 0.3 Å^−^^1^. Samples were introduced into a 2 × 2 liquid holder mounted on the Variostage sample holder (Anton Paar GmbH), with temperature regulation maintained at 25 °C *via* an integrated Peltier control unit. Data acquisition parameters for the 5v/v% Triton, 5T, 5T‐0.5S, and 5T‐1S systems included a total exposure time of 180 s, distributed across 15 frames. In contrast, for the 5T‐5S and… systems, a total exposure time of 180 s was distributed across 40 frames to enhance signal reliability. The resulting two‐dimensional scattering patterns were radially averaged to yield 1‐D scattering profiles using SAXS analysis v. 4.50.0 (Anton Paar). Background scattering was subtracted from the raw data prior to analysis.

Data processing and model fitting were conducted using the SasView 6.0 software package. It is important to note that the reported scattering intensities are not presented on an absolute scale; therefore, subsequent quantitative interpretation is provided as an approximation.

(3) I(q) ≃ P(q)S(q)

where P(q) is the form factor and S(q) is the structure factor. The C_8–10_G_1–2_ was modeled considering the following scattering length densities (SLDs): 5.68.10^−6^ Å^−2^ for the hydrophobic tail, 12.96.10^−6^ Å^−2^ for hydrophilic head and 9.44.10^−6^ Å^−2^ for the continuous phase (water). Two form factors were used: a core–shell sphere model,^[^ ^43^ ^]^ *P*_CSS_ *(q)* and a core–shell bicelle model,^[^ ^44^ ^]^ *P*_CSB_ *(q)*. A log‐normal distribution was utilized to describe the particle size dispersity, quantified by the polydispersity index, PDI. Interparticle correlations were described by a sticky hard sphere structure factor, *S(q)* ^[^ ^45^ ^]^ when appropriate.

## Conflict of Interest

The author declares no conflict of interest.

## Supporting information

Supplementary Material

## Data Availability

Data available on request from the authors.
