# Supplementary material for: Salt‐Induced Modulation of Self‐Assembly in C8‐10 AlkylPolyGlucoside/Fatty Alcohol Formulations
Source: Chempluschem. 2025 Aug 19;90(10):e202500390. doi: 10.1002/cplu.202500390 (PMC12509474; doi:10.1002/cplu.202500390)
Supplement: Supplementary file 1 — Supplementary Material [file CPLU-90-e202500390-s001.pdf]

Supporting Information

Effects of salt on a C_{8-10} AlkylPolyGlycoside Fatty alcohol formulation

L. Veronico[1*], L. Gentile[1,2]

[1] *Department of Chemistry, University of Bari Aldo Moro, via Orabona 4, Bari, Italy*
[2] *Center for Colloid and Surface Science (CSGI), via della Lastruccia 3, Florence, Italy*

^{1}H Nuclear Magnetic Resonance (NMR)

Figure S1 displays the ^{1}H NMR spectrum of Triton® CG-110 at 1 v/v%, employed to estimate the relative proton distribution between the alkyl chain and the glycosidic head group. Analysis of the signal corresponding to the terminal methyl ($-CH_3$) protons indicates that the dominant species is C_8G_1. However, due to the presence of possible alcohol impurities and the polydisperse nature of the surfactant, the sample is herein referred to as a $C_{8-10}G_{1-2}$ mixture.

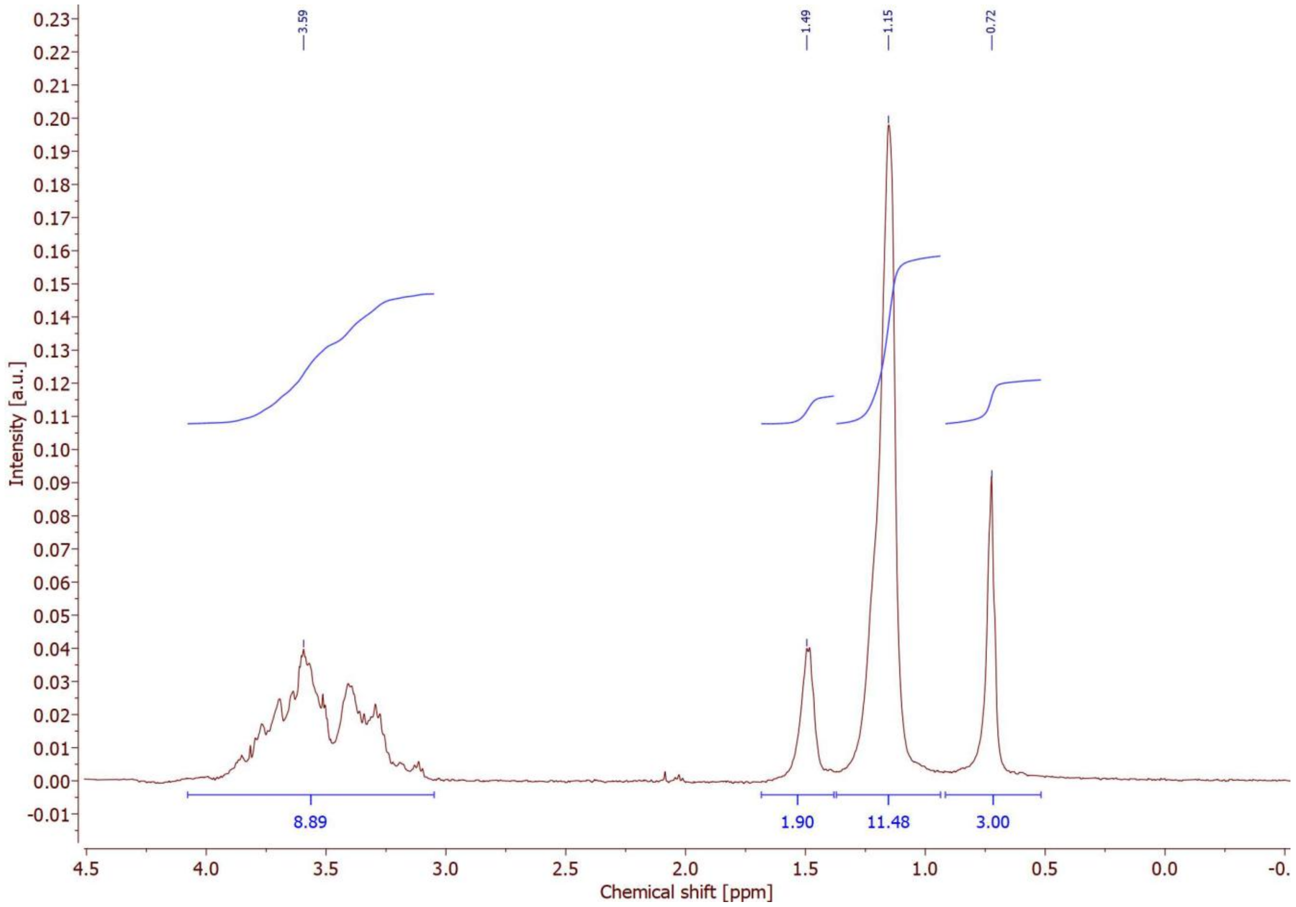

Small-Angle X-Ray Scattering (SAXS)

The small angle scattering models adopted in the main text are reported here. The core-shell sphere model adopted for 5v/v% Triton sample

$$P_{CSS}(q) = A\left\{\frac{3}{V_s}\left[V_c(\rho_c - \rho_s)\frac{sin(qR_c) - qR_c cos(qR_c)}{(qR_c)^3}V_s(\rho_s - \rho_{solv})\frac{sin\big(q(R_c + R_t)\big) - qR_t cos\big(q(R_c + R_t)\big)}{\big(q(R_c + R_t)\big)^3}\right]\right\}^2 + B \qquad (S1)$$

where A is a scale factor, V_s is the volume of the whole particle, V_c is the volume of the core, R_c is the radius of the core, R_t is the shell thickness, ρ_c is the scattering length density (SLD) of the core (hydrophobic tail) equal to $5.68\cdot10^{-6}$ 1/Å^2, ρ_s is the SLD of the shell (hydrophilic head) equal to $12.96\cdot10^{-6}$ 1/Å^2, ρ_{solv}, is the SLD of the solvent equal to $9.44\cdot10^{-6}$ 1/Å^2, while B is the background. Here, the polydispersity was applied using a lognormal distribution on both R_c and R_t.

The core-shell bicelle model adopted for 5T, 5T-0.5S, 5T-1S and 5T-5S SAXS profiles is

$$P_{CSB}(q) = \frac{A}{V_s}\left\{\left[V_c\big(\rho_c - \rho_f\big)\frac{2J_1(qRsin\alpha)}{qRsin\alpha}\frac{sin(q(L/2)cos\alpha)}{q(L/2)cos\alpha} + V_{c+f}\big(\rho_f - \rho_r\big)\frac{2J_1(qRsin\alpha)}{qRsin\alpha}\frac{sin(q(L/2 + t_F)cos\alpha)}{q(L/2 + t_F)cos\alpha} + V_s(\rho_r - \rho_{solv})\frac{2J_1(q(R + t_R)sin\alpha)}{q(R + t_R)sin\alpha}\frac{sin(q(L/2 + t_F)cos\alpha)}{q(L/2 + t_F)cos\alpha}\right]\right\}^2 sin\alpha + B \qquad (S2)$$

where A is a scale factor, V_s is the volume of the bicelle, V_c the volume of the core, V_{c+f} the volume of the core plus the volume of the faces, R is the radius of the core, L the length of the core, t_F the thickness of the face, t_R the thickness of the rim and J_1 the usual first order Bessel function. ρ_c, is the core SLD, ρ_f, the face SLD, ρ_r, the rim SLD. Here, the polydispersity was applied using a lognormal distribution on R_c.

For the SAXS profile $P_{CSB}(q)$ was adopted along with the sticky structure factor $S(q)$. The sticky hard sphere (SHS) model provides a framework for describing the structural properties of systems where particles exhibit both hard-core repulsion and a short-range attractive interaction, commonly referred to as "stickiness." This attractive component modifies the effective interparticle potential, thereby influencing the system's structure factor. Within the framework of the Percus-Yevick (PY) approximation for hard spheres, the SHS structure factor can be formulated by incorporating an additional term that accounts for this attractive interaction. The Baxter model is typically employed to implement this sticky interaction within theoretical treatments.

$$S_S(q) = \frac{1}{1 - n\cdot\hat{c}(q)} \qquad (S3)$$

where n is the number density of the particles, $\hat{c}(q)$ is the Fourier transform of the direct correlation function, which in the case of sticky hard spheres includes contributions from both hard sphere and

attractive sticky interactions. The Baxter model incorporates a "stickiness parameter", s, to account for short-range attractive interactions. This parameter is related to the depth and range of the attractive potential, with lower values of s corresponding to a stronger attraction between particles. The direct correlation function for sticky particles $c(r)$, used in calculating $S(q)$, can be written as:

$$c(r) = \begin{cases} c_{HS}(r) & for\, r > D \\ -\dfrac{A}{\delta(r-D)} & for\, r = D \end{cases} \qquad (S4)$$

where D is the particle diameter, A is related to the stickiness parameter $C_{HS}(r)$ is the particle correlation function. In the same manner this can be applied to a generical particle having a non-spherical shape.
